# Leveraging men’s education as an effective pathway for improving diet quality: Evidence from rural India

**DOI:** 10.1371/journal.pone.0283935

**Published:** 2023-11-16

**Authors:** Naveen Sunder, Soumya Gupta, Prabhu L. Pingali

**Affiliations:** 1 Department of Economics, Bentley University, Waltham, MA, United States of America; 2 Tata- Cornell Institute for Agriculture and Nutrition, Cornell University, Ithaca, NY, United States of America; Banaras Hindu University, INDIA

## Abstract

Investing in nutrition sensitive sectors such as education can be an effective strategy for combatting malnutrition. In this paper we analyze the role that men’s education plays in determining dietary diversity outcomes using primary data from 3600 households across four districts of India. Dietary diversity scores were calculated to reflect the quality of food intake, for households and women. Men’s education level was considered as the primary driver of diet diversity. To establish a causal link between men’s education and diet diversity, the education level of parents and siblings were used as instrumental variables. We find that men’s education levels are associated with significantly higher diet diversity scores both for the household and for women. The role of men’s education continues to be a significant determinant of diet quality after controlling for household and individual- level confounding factors including the education level of the woman. The results are consistent across different definitions of the diet diversity score and reference period. Methodologically we extend the evidence on the education–nutrition pathway from being associational to causal in nature. Results from this study point to the benefits of leveraging men’s education as an effective pathway for improving nutritional outcomes within households.

## Introduction

Poor quality diets are responsible for the biggest burden of disease, globally [1]. Many different policies have been implemented to combat malnutrition–in this paper, we investigate the role that education plays in addressing nutritional challenges [2]. In doing so, we add to the scant literature exploring the causal pathway between education and improved diet diversity. Since the bulk of the literature focuses on women’s education, even lesser is known about the role that *men’*s education plays in determining dietary diversity. In this paper we use primary data from 3600 households across rural India to analyze the role that men’s education can play in determining dietary diversity of not just their household, but more specifically for female members.

That socioeconomic factors such as education- particularly women’s education—can play a role in combatting malnutrition is well documented [3–7]. However, the evidence related to the impact of men’s education on dietary diversity outcomes within the household is scarce. Some studies have shown that the education of the household head is significantly associated with higher levels of diet diversity [3, 8–10]. In these studies, education hasn’t been the central focus of the analysis, and is included as a potential pathway of interest. Furthermore, it is not clarified what the gender of the household head is.

Related to the above, we know very little about whether men’s education levels affect the diet diversity at the household level differently from that at the individual level. Koppmair, Kassie and Qaim [10] find that education of the head of the household is a significant determinant of diet diversity for both, the household and the women, while both Muthini, Nzuma and Qaim [11] and Ochieng, Afari- Sefa, Lukumay and Dubois [12] conclude that while the education level of the household head is significant for household diet diversity, it is not so for women’s diet diversity. Ambikapathi et al [13] conclude that men’s education has an additive effect on household as well as women’s diets, over and above that of women’s awareness and education levels. These analyses have however used a different approach from ours. While they have established a strong association, our focus in this paper is to identify a causal pathway between men’s education and dietary diversity for women and households. Being able to strengthen this evidence base is important in light of the growing evidence of differences in dietary diversity scores for women as compared to their households [14]. And finally, understanding the role of men’s education in determining dietary diversity can inform the design of policies in a way that acknowledges the gendered impact of one of the basic drivers of malnutrition, that is education.

## Methods

### Sites and data

The data for this analysis comes from a two-round panel survey carried out by the Tata- Cornell Institute for Agriculture and Nutrition at Cornell University, for its program on Technical Assistance and Research on Indian Nutrition and Agriculture (TARINA). The TARINA surveys were conducted in four districts of India: Munger (Bihar), Maharajganj (Uttar Pradesh), Kalahandi (Odisha), and Kandhamal (Odisha). The main results presented here utilize data from the TARINA baseline survey that was conducted in 2017. The data from the midline, that was collected in 2019, is used for the purpose of robustness checks. These surveys were approved by the institutional review board at Cornell University. The approval included the recording of informed consent orally and the same was recorded in electronic tablets that were used for the survey. Furthermore, approvals were also obtained from village- level governing bodies (known as *panchayats*) before the rollout of the surveys in every village. Additional information regarding the ethical, cultural, and scientific considerations specific to inclusivity in global research is included in the supporting information (S1 Checklist)”

Each round of the survey was implemented across a total of 120 villages using a two-step sampling strategy. In the first step, 30 villages were selected in each district based on population size. In the second step, 30 households per village were selected randomly. This random selection of households was based on a census of all households that was conducted in the sample villages. The total sample size in each survey round was 3600 households.

From each household, an index man and index woman were identified as the main respondents for the survey. These women and men are chosen such that they represent the female and male decision makers in the household (and are most knowledgeable about different aspects of the household). Women who were pregnant, lactating or did not fall in the reproductive age group (15–49 years) at the time of baseline were not considered as the index woman.

The index male was asked questions related to household demographics, socioeconomic status, agricultural crop- ping practices, land use, and livestock ownership. The index woman responded to questions related to food access, Infant and Young Child Feeding practices, Water Sanitation and Hygiene, group membership, and empowerment in agriculture. The women were also administered a module on individual and household-level dietary intake over the past 24 hours and frequency of food intake over the past 7 days.

### Construction of key variables

The key variables of interest in this analysis are education and diet diversity. Education is measured in terms of the number of (completed) years of education. Our independent variable is education level of the index man. This is instrumented with the education level of his parents.

Dietary diversity scores are estimated separately for women and their households. These were constructed from detailed data on consumption of food items by the woman and the household. The list of food items was generated in consultation with community members and local implementing partners as part of the qualitative research activities that preceded the surveys. Respondents were asked about all the foods that they consumed, including food consumed away from home.

We use the standard methodology of the Food and Agriculture Organization (FAO) to calculate dietary diversity scores as the sum of food groups consumed by the woman (or household) in the previous 24- hours. In the main results the DDS is based on the ten food groups that are used to construct the Minimum Diet Diversity for Women (MDDW) score: grains, pulses, nuts and seeds, dark green leafy vegetables, Vitamin A- rich fruits and vegetables, other fruits, other vegetables, dairy, eggs, meat/ fish/ poultry [15]. To test the robustness of our results we also compute the DDS based on 12 food groups as well. The score based on 12 food groups is usually used to compute a household- level diet diversity score. The 12 food groups are grains, tubers, legumes/ nuts/ seeds, fruits, vegetables, dairy, eggs, meat, fish, oils/ fats, sweets, spices/ condiments/ beverages.

We focus on dietary diversity of both, women, and households to assess if, and how, the education–nutrition pathway plays out differently at the household level as compared to the individual- level. Usually, the diet diversity score constructed for households consists of 12 food groups while that for women consists of 10 food groups. This difference in the number of food groups prevents us from being able to compare the scores for the household with those of women. Since our aim is to look at the differential impact of men’s education on both, the household’s and the woman’s diet diversity score, a meaningful comparison is only possible when the same food groups are used to compute the diet diversity score for both. Therefore, we use the same food groups to compute both the scores. Our approach to homogenize the scales follows the approach in [14].

### Empirical strategy

#### Base specification

We first explore the association between education and diet diversity using fixed effects specifications which take the following form:

$$DD_{ihvd}=\beta_{M}\;MaleEdu_{ihvd}+\mu \;HH_{hvd}+\gamma \;Individual_{ihvd}+\pi_{village}+\varepsilon_{ihvd}$$ (1)

where, *DD*_*ihvd*_ represents the dietary diversity score (women or household DDS) of individual *i* in household *h* in village *v* belonging to district *d*, and *MaleEdu*_*i*_ are the number of years of education of the index male in the household in which individual *i* resides. The set of household- level controls (HH_hvd_) used in this specification includes production diversity of the household, categorical variables (dummy variables) for religion (Hindu or not), caste group (scheduled caste, scheduled tribe or other backward castes), and having a below poverty line (BPL) card. Individual- level factors (Individual_ihvd_) such as ages of the index woman and index man are also controlled for. We also include village fixed effects (*π*_*village*_) to control for time-invariant village-level factors. The standard errors are clustered at the village level.

Although the regressions outlined above control for a variety of individual, household and village level factors, the coefficient estimates we obtain from these fixed effects specifications are likely biased. This is because the main coefficient of interest (*β*_*M*_) is endogenous due to the existence of omitted factors that are correlated with both education and diet diversity that may not be controlled for in the regression (which are then part of the error term). To correct for this bias, we use instrumental variable (IV) regressions where we instrument for years of education using the parents’ years of education. In particular, we use the education level of the father of the index man as an IV for the education level of the index man. The specification takes the following form:

$$MaleEdu_{i}=\mu_{1}\;FathersEdu_{i}+\mu_{2}\;HH_{h}+\mu_{3}\;Individual_{i}+\pi_{village\;}+\theta_{ihv}$$ (2)

$$DD_{ihv}=\partial_{1}\;MaleEd{\;}_{i}+\partial_{2}HH_{h}+\partial_{3}\;Individual_{i}+\pi_{village}+\varepsilon_{ihv}$$ (3)

where (2) represents the first stage equation where the endogenous variable (years of education) is regressed on the instrumental variable, *FathersEdu*_*i*_, and (3) is the second stage of the Instrumental variable two stage least squares regression (IV-2SLS). The controls are the same as those described above, and the standard errors are clustered at the village level. We run an additional set of regressions with an expanded set of controls that includes presence of kitchen garden (binary), presence of livestock (binary), land ownership (acres), primary occupation of index man, and monthly food expenditures (as a proxy for income). We find that even after the inclusion of these additional controls, our results for men’s education do not change significantly. These additional regression results are included as supplementary material (see S2 Checklist).

All analysis was done using STATA 14 SE.

### Instrumental variables (IV)

The intuition behind this IV is that the education of an individual is correlated with their parents’ education level, however it is unlikely to have an effect on the woman (or her household’s) current diet diversity. This is likely because the woman lives in a different household from her parents after marriage. This is akin to the approach taken in Le and Nguyen [16] who exploit the differences in education levels of biological sisters to study the impact of maternal education on child health. Below we discuss in more detail the inclusion and exclusion criteria of this IV.

The inclusion restriction requires that the IV must be strongly correlated with the endogenous variable. In this case, it translates into checking if the IV (index man’s father’s education) is strongly associated with the endogenous variable (education of index man). We verify this using the first stage equation of the IV-2SLS strategy. The results from this exercise are presented in columns 1–3 of **Table 1**. The findings suggest that when fathers have one higher year of education, then the index man has 0.11–0.15 additional years of education. These findings are statistically significant at the one percent level. Additionally, there is no weak instruments problem—the F-statistic for these instruments is well above the threshold value of 10.

**Table 1:** Validity of instrumental variable: Impact of parental education on education of Index Man^1^.

VARIABLES	(1)	(2)	(3)
Education level of index man’s father	0.153***	0.137***	0.110***
	(0.012)	(0.010)	(0.011)
Observations	2,589	2,589	2,589
R-squared	0.109	0.258	0.395
Village Fixed Effects	NO	NO	YES
Controls^2^	NO	YES	YES

^1^ The outcome of interest is the education level of the index man. The education level of the index man’s father is used as the instrumental variable. Robust standard errors are listed in parenthesis. The standard errors are clustered at the village level. Significance levels:

*** p<0.01,

** p<0.05,

* p<0.1

^2^The control variables include the age of the index male and woman, household size and categorical variable for caste (being Hindu, scheduled caste, scheduled tribe, other backward castes), and a binary for having a kisancard.

The exclusion restriction requires that the IV must affect the outcome only through its effect on the endogenous variable (the exclusion restriction). In our case, this implies that parents’ education must affect household diet diversity only through its effect on their children’s education, and not through other channels. This is not directly testable. We postulate that the exclusion restriction is likely to hold for women, as the women live in different households from their parents (as majority of the women are married and have moved to the husband’s family). Therefore, the parents of the women are less likely to directly affect their current diet diversity through other channels.

Our approach of accounting for both men’s education (using an IV) and women’s education (without an IV) is in line with a bulk of the literature that examines the effect of women’s education on a variety of outcomes. We find that even when authors have leveraged natural experiments there are limitations to being able to control for assortative matching. As an example, Grepin and Bhardwaj [17] show the negative effect of women’s education on infant mortality in Zimbabwe using a schooling reform. In this case they have no controls for the spouse’s education in their main specification (spouses could have benefitted from the reform which could then lead to the observed effects). In fact, they consider spousal quality to be a mechanism for the effects they find, like our analysis.

However, the exclusion restriction of the IV becomes weaker in the case of men’s education, as his parents may still be living in the same household as him. If this were the case, then the education level of the parents of the index male might affect the household’s (and women’s) diet diversity through wealth/income, perceptions, control over household resources etc. This would then negate the exclusion restriction. This concern is partially assuaged by our use of an extensive set of household and individual level controls. Also, we conduct a number of checks and robustness analyses to further reduce these concerns like excluding households where the index man resides with his parents. We also test our hypotheses by using an alternate IV, one that takes into account the average education levels of the index man’s siblings.

## Results

### Descriptive statistics

Descriptive statistics for the sample are presented in **Table 2.** The mean diet diversity of households in our sample was 4.5 food groups, out of ten. This was greater than that for women (4.33 food groups out of ten). The index man and woman on average had 5.09 and 2.25 years of education respectively. Fathers of index men on average had 2.5 years of education. Hindus constituted 95% of the sample. A little over one- fifth of the sample households belong to scheduled castes while around 40% belonged to each of two other groups–scheduled tribes and other backward caste groups. The average household size was 5 and 14% of households had a kisan credit card.

**Table 2:** Descriptive statistics.

	Obs	Mean	Std. Dev.	Min	Max
Household DDS* (10 groups)	2589	4.53	1.90	0.00	10.00
Individual DDS* (10 groups)	2589	4.33	1.88	0.00	10.00
**Index Woman Education (years)**	2589	2.25	1.78	1.00	10.00
**Index Man Education (years)**	2589	5.09	4.62	0.00	19.00
**Index man’s father’s education (years)**	2589	2.50	3.85	0.00	20.00
**Hindu (% households)**	2589	0.95	0.23	0.00	1.00
**Scheduled caste (% households)**	2589	0.23	0.42	0.00	1.00
**Scheduled tribe (% households)**	2589	0.34	0.47	0.00	1.00
**Other Backward Castes (% households)**	2589	0.39	0.49	0.00	1.00
**Mahadalit (% households)**	2589	0.03	0.17	0.00	1.00
**Kisan Credit Card (% households)**	2589	0.14	0.35	0.00	1.00
**Average household size**	2589	5.01	2.02	1.00	26.00
**Age of women in completed years**	2589	37.74	11.16	1.00	84.00

*DDS refers to diet diversity score.

### Men’s education and diet diversity

Men’s education levels are associated with significantly higher diet diversity scores. At the household level, diet diversity scores are higher by 0.17 food groups for every unit increase in average education levels of men (column 1, **Table 3**). Although marginally smaller in magnitude, men’s education levels are associated with an increase in women’s diet diversity scores by 0.16 food groups (column 1, **Table 4**). The role of men’s education continues to be a significant determinant of diet quality after the inclusion of various household and individual- level confounding factors including the education level of the woman (columns 2–4 in Tables 3 and 4). If anything, the magnitude of the association increases even as controls and fixed effects are accounted for. For instance, a unit increase in men’s education level results in an increase in household diet diversity by 0.27 food group (column 4, Table 3) while women’s diet diversity scores increase by nearly 0.30 food groups (column 4, Table 4). We also note that women’s education levels are not a significant determinant in any of the models for either household or individual- level diet diversity–when controlled for in conjunction with men’s education levels.

**Table 3:** Impact of education on household diet diversity^1^.

VARIABLES	(1)	(2)	(3)	(4)
Index Male Education	0.20***	0.19*	0.33***	0.36***
	(0.06)	(0.10)	(0.11)	(0.12)
Index Women Education		0.01	-0.02	-0.01
		(0.02)	(0.02)	(0.02)
Observations	2,589	2,589	2,589	2,589
R-squared	0.008	0.010	-0.044	-0.041
Village fixed effects	NO	NO	YES	YES
Controls^2^	NO	NO	NO	YES

^1^ The outcome is the household diet diversity score (0–10) which is a count of the number of food groups consumed by atleast one member of the household in the last 24 hours. The index man’s education level is instrumented with his father’s education level. Robust standard errors are listed in parenthesis. The standard errors are clustered at the village level. Significance levels:

*** p<0.01,

** p<0.05,

* p<0.1

^2^The control variables include the age of the index male and woman, household size and categorical variable for caste (being Hindu, scheduled caste, scheduled tribe, other backward castes), and a binary for having a kisancard.

**Table 4:** Impact of education on women’s diet diversity^1^.

VARIABLES	(1)	(2)	(3)	(4)
Index Male Education	0.17***	0.15	0.32***	0.360**
	(0.062)	(0.097)	(0.102)	(0.117)
Index Women Education		0.011	-0.018	-0.013
		(0.019)	(0.018)	(0.016)
Observations	2,589	2,589	2,589	2,589
R-squared	0.006	0.010	-0.052	-0.051
Village fixed effects	NO	NO	YES	YES
Controls^2^	NO	NO	NO	YES

^1^ The outcome of interest is the woman’s diet diversity score (0–10) which is the number of food groups she consumed in the previous 24 hours. The index man’s education level is instrumented with his father’s education level. Robust standard errors are listed in parenthesis. The standard errors are clustered at the village level. Significance levels:

*** p<0.01,

** p<0.05,

* p<0.1

^2^The control variables include the age of the index male and woman, household size and categorical variable for caste (being Hindu, Scheduled caste, scheduled tribe, other backward castes), and a binary for having a kisancard.

### Robustness checks

The significant relationship between men’s education levels and diet diversity presented in the preceding section is consistent even when we modify the empirical strategy. To begin with we run the same specifications for an alternate dataset–for the same set of households—that comes from the TARINA midline survey. This accounts for any changes in household food access and availability at a different point in time. We also use a different definition of the diet diversity score, one that is based on the 12- food groups of the Household Diet Diversity Score (**S1 Table)**. By doing so we can check if the relationship depends on how the food groups are aggregated to compute the diet diversity score. Yet another modification is the reference period used to calculate the diet diversity scores. Although the main results are based on a 24- hour recall period, we also compute diet diversity based on a 7- day recall for both, 10 food groups (**S2 Table**) and 12 food groups (**S3 Table**). This allows us to capture variations in day-to-day consumption, at the individual as well as household level. And finally, we alter the functional form of the regressions by using both poisson and negative binomial regressions. Across all these modifications, men’s education continues to be a significant determinant of diet diversity, indicating that this pattern is certainly present in the data.

### Validity of the IV strategy

Earlier we had discussed the concern that the exclusion restriction for the IV strategy may not be satisfied in the case of men’s education since the index male’s parents may also reside in the same household as him, and hence have a direct effect on the diet diversity. To reduce concerns that such a situation may be driving our results, we consider two different checks. In the first check, we drop households where the parents of the index male live in the same household as the man. There are ~600 households of this type in our dataset. Therefore, the remaining sample consists of households where the parents of the index male are not in the same household as him and are hence not likely to have a direct effect on their diet diversity. We re-estimate our main results with this sample in **Table 5** and find that the pattern of results obtained are similar to the main results reported in section 3.2. Moreover, these findings persist even when the definition of the diet diversity scores is changed to one based on 12 food groups (**S4 Table).**

**Table 5:** Impact of education on diet diversity of women and their households after removing households with co-resident parents^1^.

	Household diet diversity score				Women’s diet diversity score			
	(1)	(2)	(3)	(4)	(5)	(6)	(7)	(8)
Index Male Education	0.17**	0.14	0.29***	0.32***	0.17**	0.13	0.28***	0.32***
	(0.07)	(0.11)	(0.11)	(0.12)	(0.07)	(0.10)	(0.10)	(0.12)
Index Women Education		0.02	-0.01	-0.00		0.02	-0.01	-0.00
		(0.02)	(0.02)	(0.02)		(0.02)	(0.02)	(0.02)
Observations	2,167	2,167	2,167	2,167	2,167	2,167	2,167	2,167
R-squared	0.009	0.013	-0.029	-0.026	0.006	0.011	-0.037	-0.037
Village fixed effects	NO	NO	YES	YES	NO	NO	YES	YES
Controls^2^	NO	NO	NO	YES	NO	NO	NO	YES

^1^ The outcome of interest is the diet diversity score. The household (or woman) diet diversity score (0–10) is a count of the number of food groups consumed by the household (or woman) in the previous 24 hours. The index man’s education level is instrumented with his father’s education level. Robust standard errors are listed in parenthesis. The standard errors are clustered at the village level. Significance levels:

*** p<0.01,

** p<0.05,

* p<0.1

^2^The control variables include the age of the index male and woman, household size and categorical variable for caste (being Hindu, Scheduled caste, scheduled tribe, other backward castes), and a binary for having a kisancard.

In another potential sensitivity check, we use a different set of IVs for men’s education. Here, we propose the use of the average education level of his siblings as instrumental variables. This is more likely to satisfy the exclusion restriction as the siblings are much less likely to be co-resident. Looking at the results using siblings’ education as IV for men’s education (**Table 6**), we again find that the pattern of findings in Tables 2 and 3 are unaffected for both household and women’s diet diversity. As a further check, we replicate this analysis after removing households that have co-resident siblings (~150 households)–the results still stay robust after this change (see **Table 7**).

**Table 6:** Impact of education on diet diversity (IV for male education = his siblings’ education)^1^.

	Household diet diversity score				Women’s diet diversity score			
	(1)	(2)	(3)	(4)	(5)	(6)	(7)	(8)
Index Male Education	0.33***	0.28***	0.30***	0.34***	0.23***	0.20***	0.25***	0.29***
	(0.06)	(0.09)	(0.09)	(0.12)	(0.05)	(0.07)	(0.07)	(0.09)
Index Women Education		0.02	0.02	0.02		0.01	0.01	0.01
		(0.02)	(0.02)	(0.02)		(0.01)	(0.01)	(0.01)
Observations	2,693	2,693	2,693	2,693	2,693	2,693	2,693	2,693
R-squared	0.006	0.013	0.001	0.003	0.002	0.006	-0.014	-0.013
Village fixed effects	NO	NO	YES	YES	NO	NO	YES	YES
Controls^2^	NO	NO	NO	YES	NO	NO	NO	YES

^1^ The outcome of interest is the diet diversity score. The household (or woman) diet diversity score (0–10) is a count of the number of food groups consumed by the household (or woman) in the previous 24 hours. The index man’s education level is instrumented with his siblings’ education level. Robust standard errors are listed in parenthesis. The standard errors are clustered at the village level. Significance levels:

*** p<0.01,

** p<0.05,

* p<0.1

^2^The control variables include the age of the index male and woman, household size and categorical variable for caste (being Hindu, Scheduled caste, scheduled tribe, other backward castes), and a binary for having a kisancard.

**Table 7:** Impact of education on diet diversity^1^ (IV for male education = his siblings’ education, excluding households with co-resident siblings).

	Household diet diversity score				Women’s diet diversity score			
	(1)	(2)	(3)	(4)	(5)	(6)	(7)	(8)
Index Male Education	0.34***	0.28***	0.31***	0.36***	0.23***	0.19**	0.25***	0.29***
	(0.07)	(0.10)	(0.10)	(0.12)	(0.06)	(0.08)	(0.08)	(0.09)
Index Women Education		0.03	0.03	0.03		0.02	0.01	0.02
		(0.02)	(0.02)	(0.02)		(0.01)	(0.01)	(0.01)
Observations	2,257	2,257	2,257	2,257	2,257	2,257	2,257	2,257
R-squared	0.004	0.013	0.001	0.003	0.001	0.008	-0.013	-0.011
Village fixed effects	NO	NO	YES	YES	NO	NO	YES	YES
Controls^2^	NO	NO	NO	YES	NO	NO	NO	YES

^1^ HH refers to household, DD refers to diet diversity. The household (or woman) diet diversity score (0–10) is a count of the number of food groups consumed by the household (or woman) in the previous 24 hours. The index man’s education level is instrumented with his siblings’ education level. Robust standard errors are listed in parenthesis. The standard errors are clustered at the village level. Significance levels:

*** p<0.01,

** p<0.05,

* p<0.1

^2^The control variables include the age of the index male and woman, household size and categorical variable for caste (being Hindu, Scheduled caste, scheduled tribe, other backward castes), and a binary for having a kisancard.

## Discussion and conclusion

In this paper we establish a causal relationship between men’s education level and diet diversity for women and their households. We show that men’s education is a significant determinant of diets at the household and intrahousehold level. In households where men have a higher number of years of education, diets are diverse for both the women as well as other members of the household. This relationship continues to remain positive and significant even after the inclusion of other household and individual- level factors, including the education level of women (which by itself is not a significant variable). The latter contrasts with findings from recent studies that have explicitly account for men’s education together with that of women. For example, Chegere and Stage [18] control for both, education of household head and the education level of the highest educated woman in the household. They find that both these factors are significant determinants of household diet diversity in Tanzania.

The role of education in shaping health outcomes is well- recognized. Much of the evidence in support of this has focused on the educational attainment of women and children. Women’s education can influence health-related behaviors and practices–more educated women might take better care of themselves and the people around them. Rammohan, Pritchard, Dibley and Vicol [5] find that education is associated with better diet diversity amongst pregnant women, and that women with poor diet diversity had low birth weight babies. Similarly, dietary intake of pregnant women was more diverse, the better educated the women were [7] while that of children was higher for more educated household head [19]. Women’s education has also been a significant determinant of their own diet diversity in Bangladesh [4].

In comparison to women’s education, relatively less attention has been given to the role of men’s education in determining diet diversity. The education level of the head of the household has been associated with significantly higher household dietary diversity in other parts of India such as the semi- arid regions of Telangana and Maharashtra [3]. The head of the household is likely to be make in these contexts, but this has not been clearly explored by these studies. Our results point to a strong relationship between men’s education and diet diversity for women and their households. Furthermore, this relationship is consistent when the dataset, scale and reference of the DDS is modified.

Methodologically a significant chunk of the evidence on the education–nutrition pathway has been associational in nature. For instance, men’s (paternal) education has been identified as a strong predictor of child nutrition outcomes in various settings [20–24]. The evidence points to a growing realization that men need to be included in policy design to take account ‘societal issues and intrahousehold power relations’ [24]. However, this study is one of the first to explore the plausibly causal effects of men’s education on diet outcomes for households and women. While recent literature has shown a positive association between education of the household head and diet diversity, such evidence has stopped short of identifying a causal pathway [8–10, 25] We use an IV technique to document a causal relationship between education and nutrition. We instrument men’s education first using education levels of their father. Our results point to a men’s education levels being a strong predictor of diet diversity. This relationship is consistent even when we use an alternate IV, namely the average education level of the man’s siblings.

We acknowledge the limitations of the use of father’s education as an instrument for men’s education in our analysis. To begin with father’s education can influence the dietary diversity outcomes through the choice of the daughter in law. Such as assortative matching in the marriage market is expected in the Indian context where marriages are ‘arranged’. In other words, more educated men are more likely to marry girls with similar educational levels. Our IV strategy does not account for the effect of such an assortative matching. However, we assuage some of these concerns by controlling for the wife’s education (index female education) in our regressions. This controls for the *direct* effect that women have on their own diet diversity and the diet diversity of the household and should enable us to look at the additional effect that male education has on diet diversity. Interestingly what we find is that men’s education continues to remain a significant determinant of diet diversity even after the inclusion of the woman’s education level.

Furthermore, parents’ education is likely to influence the diet diversity that the index man grew up with, and in many case the daughter- in- law would continue to maintain those food preferences there by influencing diet diversity of the household and herself. This can be the case even if the index man and woman do not co- reside with the index man’s parents. And finally, we are aware that the external validity of our results is limited in as much as the analysis relies on survey data from 4 districts from 3 states in India. The strength of the relationship between men’s education and diet diversity is likely to vary within the country depending on differences in levels of economic growth and development. Having said that, our study areas are some of the poorest districts in the country with a persistent burden of malnutrition, especially amongst women and children and bring to the fore the importance of leveraging education as an important nutrition- sensitive intervention for improving nutritional outcomes.

The discourse on intrahousehold food allocation in South Asia has focused on factors such as incomes, bargaining power, social status, food behaviors, tastes, preferences, and interpersonal relationships [26]. These can result in differences in food intake of women as compared to men in India [14]. Such patterns have also been observed in other south Asian countries, like Nepal [27] and Bangladesh [28]. For the most part these factors have been studied from the point of view of the woman. For instance, women’s social status or bargaining power or nutrition knowledge and empowerment have all been identified as important drivers of nutritional outcomes for women, their children and their households. Equitable food allocation within the household however cannot occur by accounting only for the, unquestionably important, role played by women as gatekeepers of their household’s nutrition. Equally crucial to the discussion is the role that men play in household food security as fathers, husbands, income earners and key decisionmakers [23, 29]. The results presented in this paper reflect the fact that any strategy for improving nutritional outcomes for households must bring men into the fold even as the focus is on women.

## Supporting information

S1 Checklist Inclusivity in global research. (PDF)

S2 Checklist Results with expanded set of controls. (PDF)

S1 Dataset File containing minimal dataset. (DTA)

S1 Table Using 12- food group diet diversity scores to test the relationship between men’s education and diet diversity. (PDF)

S2 Table Relationship between men’s education and diet diversity (10- point score) using weekly recall period. (PDF)

S3 Table Relationship between men’s education and diet diversity (12- point score) using weekly recall period. (PDF)

S4 Table Men’s education as a determinant of diet diversity, using 12- group definition of diet diversity, excluding households with co-resident parents. (PDF)
