# Supplementary material for: Leveraging men’s education as an effective pathway for improving diet quality: Evidence from rural India
Source: PLoS One. 2023 Nov 16;18(11):e0283935. doi: 10.1371/journal.pone.0283935 (PMC10653534; doi:10.1371/journal.pone.0283935)
Supplement: S2 Checklist — (PDF) [file pone.0283935.s002.pdf]

PONE-D-22-09842

Leveraging men's education as an effective pathway for improving diet quality: evidence from rural India

S2 Tables

The main regression results from the manuscript are reproduced here with an expanded set of controls that now include presence of kitchen garden (binary), presence of livestock (binary), land ownership (acres), primary occupation of index man, and monthly food expenditures (as a proxy for income).

Table A: Validity of instrumental variable: Impact of parental education on education of Index Man[1]

VARIABLES	(1)	(2)	(3)
Education level of index man's father	0.153***	0.126***	0.103***
	(0.012)	(0.009)	(0.010)
Observations	2,588	2,588	2,588
R-squared	0.109	0.293	0.415
Village FE	NO	NO	YES
Controls[2]	NO	YES	YES
Cluster	Village	Village	Village

[1] The outcome of interest is the education level of the index man. The education level of the index man's father is used as the instrumental variable. Robust standard errors are listed in parenthesis. The standard errors are clustered at the village level. Significance levels: *** p<0.01, ** p<0.05, * p<0.1

[2]The control variables include the age of the index male and woman, household size and categorical variable for being Hindu, Scheduled caste, scheduled tribe, other backward castes, binary for having a kisancard, kitchen garden and livestock; area of land owned (acres), occupation of index man, monthly total food expenditure (proxy for income).

TABLE B: Impact of education on household diet diversity[1]

VARIABLES	(1)	(2)	(3)	(4)
Index Male Education	0.204***	0.191*	0.333***	0.386***
	(0.065)	(0.104)	(0.106)	(0.123)
Index Woman Education		0.006	-0.020	-0.018
		(0.020)	(0.018)	(0.017)
Observations	2,589	2,589	2,589	2,588
R-squared	0.008	0.010	-0.044	-0.014
Village FE	NO	NO	YES	YES
Controls[2]	NO	NO	NO	YES
Cluster	Village	Village	Village	Village

[1] The outcome is the household diet diversity score (0- 10) which is a count of the number of food groups consumed by atleast one member of the household in the last 24 hours. The index man's education level is instrumented with his father's education level. Robust standard errors are listed in parenthesis. The standard errors are clustered at the village level. Significance levels: *** p<0.01, ** p<0.05, * p<0.1

[2]The control variables include the age of the index male and woman, household size and categorical variable for being Hindu, Scheduled caste, scheduled tribe, other backward castes, binary for having a kisancard, kitchen garden and livestock; area of land owned (acres), occupation of index man, monthly total food expenditure (proxy for income).

TABLE C: Impact of education on women's diet diversity[1]

VARIABLES	(1)	(2)	(3)	(4)
Index Male Education	0.177***	0.151	0.325***	0.383***
	(0.062)	(0.097)	(0.102)	(0.117)
Index Woman Education		0.011	-0.018	-0.016
		(0.019)	(0.018)	(0.016)
Observations	2,589	2,589	2,589	2,588
R-squared	0.006	0.010	-0.052	-0.029
Village FE	NO	NO	YES	YES
Controls[2]	NO	NO	NO	YES
Cluster	Village	Village	Village	Village

[1] The outcome is the women's diet diversity score (0- 10) which is a count of the number of food groups consumed by atleast one member of the household in the last 24 hours. The index man's education level is instrumented with his father's education level. Robust standard errors are listed in parenthesis. The standard errors are clustered at the village level. Significance levels: *** p<0.01, ** p<0.05, * p<0.1

[2]The control variables include the age of the index male and woman, household size and categorical variable for being Hindu, Scheduled caste, scheduled tribe, other backward castes, binary for having a kisancard, kitchen garden and livestock; area of land owned (acres), occupation of index man, monthly total food expenditure (proxy for income).

TABLE D: Impact of education on diet diversity of women and their households after removing households with co-resident parents[1]

VARIABLES	Household diet diversity score				Women's diet diversity score			
	(1)	(2)	(3)	(4)	(5)	(6)	(7)	(8)
Index Male Education	0.18***	0.14	0.29***	0.34***	0.17***	0.13	0.29***	0.33***
	(0.07)	(0.11)	(0.11)	(0.12)	(0.07)	(0.10)	(0.10)	(0.11)
Index Women Education		0.01	-0.01	-0.01		0.02	-0.01	-0.00
		(0.02)	(0.02)	(0.02)		(0.02)	(0.02)	(0.02)
Observations	2,166	2,166	2,166	2,166	2,166	2,166	2,166	2,166
R-squared	0.008	0.012	-0.032	0.005	0.005	0.010	-0.040	-0.011
Village FE	NO	NO	YES	YES	NO	NO	YES	YES
Controls[2]	NO	NO	NO	YES	NO	NO	NO	YES
Cluster	Village	Village	Village	Village	Village	Village	Village	Village

[1] The outcome of interest is the diet diversity score. The household (or woman) diet diversity score (0 – 10) is a count of the number of food groups consumed by the household (or woman) in the previous 24 hours. The index man's education level is instrumented with his father's education level. Robust standard errors are listed in parenthesis. The standard errors are clustered at the village level. Significance levels: *** p<0.01, ** p<0.05, * p<0.1

[2]The control variables include the age of the index male and woman, household size and categorical variable for being Hindu, Scheduled caste, scheduled tribe, other backward castes, binary for having a kisancard, kitchen garden and livestock; area of land owned (acres), occupation of index man, monthly total food expenditure (proxy for income).

Table E: Impact of education on diet diversity (IV for male education = his siblings' education)[1]

VARIABLES	Household diet diversity score				Woman diet diversity score			
	(1)	(2)	(3)	(4)	(5)	(6)	(7)	(8)
Index Male Education	0.163***	0.129*	0.172**	0.207**	0.16***	0.13*	0.18***	0.22***
	(0.050)	(0.070)	(0.070)	(0.087)	(0.05)	(0.07)	(0.07)	(0.08)
Index Woman Education		0.016	0.004	0.002		0.02	0.00	0.00
		(0.014)	(0.013)	(0.012)		(0.01)	(0.01)	(0.01)
Observations	2,693	2,693	2,693	2,693	2,693	2,693	2,693	2,693
R-squared	0.009	0.013	-0.004	0.032	0.006	0.010	-0.011	0.021
Village FE	NO	NO	YES	YES	NO	NO	YES	YES
Controls[2]	NO	NO	NO	YES	NO	NO	NO	YES
Cluster	Village	Village	Village	Village	Village	Village	Village	Village

[1] The outcome of interest is the diet diversity score. The household (or woman) diet diversity score (0 – 10) is a count of the number of food groups consumed by the household (or woman) in the previous 24 hours. The index man's education level is instrumented with his siblings' education level. Robust standard errors are listed in parenthesis. The standard errors are clustered at the village level. Significance levels: *** p<0.01, ** p<0.05, * p<0.1

[2]The control variables include the age of the index male and woman, household size and categorical variable for being Hindu, Scheduled caste, scheduled tribe, other backward castes, binary for having a kisancard, kitchen garden and livestock; area of land owned (acres), occupation of index man, monthly total food expenditure (proxy for income).

Table F: Impact of education on diet diversity[1] (IV for male education = his siblings' education, excluding households with co-resident siblings)

VARIABLES	Household diet diversity score				Woman's diet diversity score			
	(1)	(2)	(3)	(4)	(5)	(6)	(7)	(8)
Index Male Education	0.15***	0.12	0.18**	0.22**	0.16***	0.11	0.18***	0.21***
	(0.06)	(0.07)	(0.07)	(0.08)	(0.06)	(0.07)	(0.07)	(0.08)
Index Women Education		0.02	0.01	0.01		0.02*	0.01	0.01
		(0.01)	(0.01)	(0.01)		(0.01)	(0.01)	(0.01)
Observations	2,257	2,257	2,257	2,257	2,257	2,257	2,257	2,257
R-squared	0.007	0.011	-0.005	0.033	0.005	0.011	-0.009	0.026
Village FE	NO	NO	YES	YES	NO	NO	YES	YES
Controls[2]	NO	NO	NO	YES	NO	NO	NO	YES
Cluster	Village	Village	Village	Village	Village	Village	Village	Village

[1] HH refers to household, DD refers to diet diversity. The household (or woman) diet diversity score (0 – 10) is a count of the number of food groups consumed by the household (or woman) in the previous 24 hours. The index man's education level is instrumented with his siblings' education level. Robust standard errors are listed in parenthesis. The standard errors are clustered at the village level. Significance levels: *** p<0.01, ** p<0.05, * p<0.1

[2]The control variables include the age of the index male and woman, household size and categorical variable for being Hindu, Scheduled caste, scheduled tribe, other backward castes, binary for having a kisancard, kitchen garden and livestock; area of land owned (acres), occupation of index man, monthly total food expenditure (proxy for income).
