# Supplementary material for: Leveraging men’s education as an effective pathway for improving diet quality: Evidence from rural India
Source: PLoS One. 2023 Nov 16;18(11):e0283935. doi: 10.1371/journal.pone.0283935 (PMC10653534; doi:10.1371/journal.pone.0283935)
Supplement: S1 Table — (PDF) [file pone.0283935.s004.pdf]

Leveraging men's education as an effective pathway for improving diet quality: evidence from rural India

S1 Table. Using 12- food group diet diversity scores to test the relationship between men's education and diet diversity

VARIABLES	(1)	(2)	(3)	(4)	(5)	(6)	(7)	(8)
	HH[1]	HH[1]	HH[1]	HH[1]	Woman[2]	Woman[2]	Woman[2]	Woman[2]
Index Male Education	0.258***	0.283***	0.318***	0.349***	0.186***	0.198**	0.257***	0.283***
	(0.059)	(0.093)	(0.099)	(0.116)	(0.053)	(0.082)	(0.087)	(0.101)
Index Woman Education		-0.011	-0.012	-0.008		-0.005	-0.010	-0.007
		(0.017)	(0.016)	(0.015)		(0.015)	(0.015)	(0.014)
Observations	2,589	2,589	2,589	2,589	2,589	2,589	2,589	2,589
R-squared	-0.014	-0.022	-0.043	-0.044	-0.010	-0.014	-0.046	-0.046
Village Fixed effects	NO	NO	YES	YES	NO	NO	YES	YES
Controls[3]	NO	NO	NO	YES	NO	NO	NO	YES

[1] HH refers to household diet diversity score. [2]Woman refers to woman's diet diversity score. The household (or woman) diet diversity score (0 – 12) is a count of the number of food groups consumed by the household (or woman) in the previous 24 hours. The index man's education level is instrumented with his father's education level. Robust standard errors are listed in parenthesis. The standard errors are clustered at the village level. Significance levels: *** p<0.01, ** p<0.05, * p<0.1

[3]The control variables include the age of the index male and woman, household size and categorical variable for caste (being Hindu, scheduled caste, scheduled tribe, other backward castes), and a binary for having a kisancard.
