# Supplementary material for: Leveraging men’s education as an effective pathway for improving diet quality: Evidence from rural India
Source: PLoS One. 2023 Nov 16;18(11):e0283935. doi: 10.1371/journal.pone.0283935 (PMC10653534; doi:10.1371/journal.pone.0283935)
Supplement: S2 Table — (PDF) [file pone.0283935.s005.pdf]

Leveraging men's education as an effective pathway for improving diet quality: evidence from rural India

S2 Table. Relationship between men's education and diet diversity (10- point score) using weekly recall period

VARIABLES	(1)	(2)	(3)	(4)	(5)	(6)	(7)	(8)
	HH[1]	HH[1]	HH[1]	HH[1]	Woman[2]	Woman[2]	Woman[2]	Woman[2]
Index Male Education	0.23***	0.16	0.36***	0.38***	0.23***	0.17	0.37***	0.40***
	(0.08)	(0.12)	(0.11)	(0.13)	(0.08)	(0.12)	(0.11)	(0.13)
Index Woman Education		0.03	-0.01	0.00		0.03	-0.01	-0.00
		(0.02)	(0.02)	(0.02)		(0.02)	(0.02)	(0.02)
Observations	2,589	2,589	2,589	2,589	2,589	2,589	2,589	2,589
R-squared	0.027	0.034	-0.057	-0.034	0.019	0.025	-0.075	-0.048
Village Fixed effects	NO	NO	YES	YES	NO	NO	YES	YES
Controls[3]	NO	NO	NO	YES	NO	NO	NO	YES

[1] HH refers to household diet diversity score. [2]Woman refers to woman's diet diversity score. The household (or woman) diet diversity score (0 – 12) is a count of the number of food groups consumed by the household (or woman) in the previous 7 days. The index man's education level is instrumented with his father's education level. Robust standard errors are listed in parenthesis. The standard errors are clustered at the village level. Significance levels: *** p<0.01, ** p<0.05, * p<0.1

[3]The control variables include the age of the index male and woman, household size and categorical variable for caste (being Hindu, scheduled caste, scheduled tribe, other backward castes), and a binary for having a kisancard.
